# Supplementary material for: Determinants of the happiness of adolescents: A leisure perspective
Source: PLoS One. 2024 Apr 9;19(4):e0301843. doi: 10.1371/journal.pone.0301843 (PMC11003699; doi:10.1371/journal.pone.0301843)
Supplement: Attachment — Submitted filename: Response to reviewers.docx [file pone.0301843.s003.docx]

Dear. Dr. Henri Tilga, Academic Editor

First of all, we really appreciate your kind and strong support on the manuscript submission.

As per your and reviewers’ revision request, we revised our manuscript so that the revised version of the manuscript fully addresses the points raised during the review process.

Here are the responses and revisions.

**Title of the manuscript**

**Determinants of the Happiness of Adolescents: A Leisure Perspective**

**Data availability statement**

This study used the method of machine learning to analyze national statistical data, National Leisure Activity Survey, which is open to the public. The data used in this study were from the National Leisure Activity Survey 2019, which is a national statistic produced by the Ministry of Culture, Sports and Tourism in the Republic of Korea (Statistics approved by the National Statistics Office - approval number: No. 113014).

**Reviewer comments**

1. Enhance methodological detail and provide more detailed information about the specific machine learning techniques employed, including the choice of algorithms, feature selection, and model evaluation metrics.

Response: We added the following sections in the method section.

**Model and evaluation**

A prediction model was built based on the supervised learning algorithm of machine learning, and the performance of the model was evaluated. The process of creating a regression prediction model is a process of optimizing the coefficients of the independent variables by minimizing the difference between the predicted value and the actual value, that is, the value of the cost function. In this study, general linear regression, the most representative regression model, and regulated linear models (i.e., Ridge Regression, Lasso Regression, Elastic Net Regression), which are improvement models of general linear regression, were used. The regularized regression model is a method of increasing the weight of variables of high importance and lowering the weight of variables of low importance to increase the possibility of generalization by preventing multicollinearity between variables and overfitting of the prediction model. In addition, the ensemble model of eXtreme Gradient Boost (XGB) and Light Gradient Boosting Machine (LGBM) was used. An ensemble model is a method of predicting results by merging different prediction models. As such, a total of six machine learning algorithms (Linear Regression, Ridge Regression, Lasso Regression, Elastic Net Regression, eXtreme Gradient Boost, & Light Gradient Boosting Machine) were used in this study.

The evaluation indicators used were R² (r-squared) and RMSE (root mean square error), which are representative performance indicators of regression models. R² refers to a coefficient that measures how much the regression line estimated by the regression analysis explains the actual sample. If this value is 1, it means that the regression line is in perfect agreement with the data. Conversely, if the coefficient of determination is 0, it means that the regression line does not explain the distribution of the data at all. RMSE is a generalized measure of standard deviation and represents the average difference between the value predicted by the prediction model and the actual value. Therefore, the lower the value of root mean square error, the better the prediction model.

**Evaluation of the importance of predictor variables**

The relative importance of the predictor variables was evaluated based on the final model. The evaluation indicator used was a regression coefficient that indicates the influence of the predictor variable. Among the predictive factors entered into the model, three factors with high regression coefficients were extracted to identify the determinants of happiness.

2. Address survey limitations by acknowledging the inherent limitations of survey-based research, including potential biases, and discuss how these limitations may impact the study's findings and their generalizability.

Response: We added the limitations at the conclusion section as follows:

However, this study has some limitations from an academic standpoint. Firstly, this study used quantitative research methods. Quantitative research methods have limitations in providing an in-depth understanding of the experiences, attitudes, motivations, and contexts of the research subjects. In particular, most of the data used in this study are multiple-choice surveys, so there is a limitation in not being able to specifically express the opinions or thoughts of the subjects. Considering this, qualitative research methods also need to be considered for future studies.

3. Consider cultural context while explicitly recognizing the cultural and geographical specificity of the study and discuss how these factors may influence the observed relationships between leisure factors and adolescent happiness.

Response: We added the limitations at the conclusion section as follows:

Secondly, since this study used domestic data to fully investigate the domestic situation on the relationship between leisure factors and adolescents’ happened within the Korean context, it is difficult to generalize the results of the study internationally. Although the results and claims made in this study are supported by foreign studies which were conducted internationally and also by explanations about the domestic cultural context, it would be desirable to extend the current results with future international data analyses in future studies so that more meaningful and comprehensive findings can be derived.

4. Provide practical recommendations while clearly articulating them based on the study's findings, particularly in terms of policy implications and interventions that could improve adolescents' leisure experiences and well-being.

Response: We added the following at the conclusion section as follows:

Many countries recognize the importance of leisure activities for youth and are making related policy efforts. In this way, the findings of this research imply that rather than leaving leisure activities to individual youth, there is a need to support them institutionally at the national level. In particular, as the importance of public leisure spaces has been highlighted in this study, there is a need to expand public leisure facilities for youth.

5. Include a table of authors at the end of the literature review section to analyze the most relevant paper in each variable

Response: We added the following table at the end of the literature review section.

**Table 1. Leisure factors affecting the happiness of adolescents**

| **Factor** | **Previous studies** |
| --- | --- |
| Diversity of leisure activities | Dahan-Oliel et al(2012) [54]  Santini et al(2020) [17] |
| Type of leisure activities | Ito et al(2019) [56]  Lee et al(2017) [14]  Shin & You(2013) [18] |
| Presence or absence of leisure companions | Gray(2011) [68]  Lam & McHale(2015) [69]  Larson & Csikszentmihalyi(2014) [70]  Parker et al(2022) [16] |
| frequency of leisure activities | Doerksen et al(2014) [73]  Moljord et al(2011) [15]  Te Velde et al(2018) [71]  Toker & Kalıpçı(2021) [72] |
| Amount of leisure time | Manolis &Roberts(2012) [22]  Moore & Ohtsuka(2000) [75]  Uusitalo-Malmivaara(2014) [74]  Wang et al(2011) [24] |
| Amount of leisure cost | Kim & Kim(2020) [20] |
| Leisure space | Bailey & Fernando(2012) [21]  Leonard(2015) [81]  Weng & Chiang(2014) [82]  Benita et al(2019) [85] |
| Balance between study and leisure | Lee & Kim(2021) [87]  Ratelle et al(2005) [29] |
| Degree of leisure perception and attitude | Bailey et al(2016) [26]  Gökyürek(2016) [27]  Lepp(2018) [28] |

6. Include a diagram to illustrate the applied methodological process to your research

Response: We added a diagram to illustrate the applied methodological process at the beginning of the research method section.

Figure 1. Research procedures and methods

7. Expand your conclusions including your future work and the relevance in the managerial, academic, and government arena.

Response: We expanded our conclusions as follows.

**Conclusion**

The purpose of this study is to establish a happiness prediction model for adolescents based on machine learning, and to identify the relative importance of predictive factors to derive major leisure factors that affect the happiness of adolescents. The conclusions obtained through this study are as follows.

This study found that leisure perceptions, academic and leisure balance, and public leisure spaces all have a very important impact on adolescents' well-being. These findings will help other researchers investigating the relationship between leisure and happiness in adolescents design their studies. Furthermore, these results will have a positive impact on youth leisure policymakers. Many countries recognize the importance of leisure activities for youth and are making related policy efforts. In this way, the findings of this research imply that rather than leaving leisure activities to individual youth, there is a need to support them institutionally at the national level. In particular, as the importance of public leisure spaces has been highlighted in this study, there is a need to expand public leisure facilities for youth.

However, this study has some limitations from an academic standpoint. Firstly, this study used quantitative research methods. Quantitative research methods have limitations in providing an in-depth understanding of the experiences, attitudes, motivations, and contexts of the research subjects. In particular, most of the data used in this study are multiple-choice surveys, so there is a limitation in not being able to specifically express the opinions or thoughts of the subjects. Considering this, qualitative research methods also need to be considered for future studies. Secondly, since this study used domestic data to fully investigate the domestic situation on the relationship between leisure factors and adolescents’ happened within the Korean context, it is difficult to generalize the results of the study internationally. Although the results and claims made in this study are supported by foreign studies which were conducted internationally and also by explanations about the domestic cultural context, it would be desirable to extend the current results with future international data analyses in future studies so that more meaningful and comprehensive findings can be derived.

In addition, longitudinal studies to understand how the leisure factors that affect adolescent happiness change over time would be required. Lastly, this study has limitations in in-depth discussion of the relationship between happiness and leisure among adolescents. In this regard, in the future, it would be necessary to expand the current results through in-depth analysis according to the characteristics of adolescents, such as gender, school level, and region of residence.

Despite these limitations, it is significant that this study explored the leisure factors that determine happiness in adolescents using reliable and nationally representative national statistics. In particular, it is significant that by using machine learning, which is considered to be the core of predictive analytics, it was possible to improve the accuracy of research results. Finally, the findings of this research will serve as a basis for formulating and promoting youth leisure policies.

8. The authors need to explain further each of the six machine learning algorithms (LinearRegression, Ridge, Lasso, ElasticNet, XGBoost, and LightGBM)

Response: Response: We added the following sections in the method section.

**Model and evaluation**

A prediction model was built based on the supervised learning algorithm of machine learning, and the performance of the model was evaluated. The process of creating a regression prediction model is a process of optimizing the coefficients of the independent variables by minimizing the difference between the predicted value and the actual value, that is, the value of the cost function. In this study, general linear regression, the most representative regression model, and regulated linear models (i.e., Ridge Regression, Lasso Regression, Elastic Net Regression), which are improvement models of general linear regression, were used. The regularized regression model is a method of increasing the weight of variables of high importance and lowering the weight of variables of low importance to increase the possibility of generalization by preventing multicollinearity between variables and overfitting of the prediction model. In addition, the ensemble model of eXtreme Gradient Boost (XGB) and Light Gradient Boosting Machine (LGBM) was used. An ensemble model is a method of predicting results by merging different prediction models. As such, a total of six machine learning algorithms (Linear Regression, Ridge Regression, Lasso Regression, Elastic Net Regression, eXtreme Gradient Boost, & Light Gradient Boosting Machine) were used in this study.

The evaluation indicators used were R² (r-squared) and RMSE (root mean square error), which are representative performance indicators of regression models. R² refers to a coefficient that measures how much the regression line estimated by the regression analysis explains the actual sample. If this value is 1, it means that the regression line is in perfect agreement with the data. Conversely, if the coefficient of determination is 0, it means that the regression line does not explain the distribution of the data at all. RMSE is a generalized measure of standard deviation and represents the average difference between the value predicted by the prediction model and the actual value. Therefore, the lower the value of root mean square error, the better the prediction model.

**Evaluation of the importance of predictor variables**

The relative importance of the predictor variables was evaluated based on the final model. The evaluation indicator used was a regression coefficient that indicates the influence of the predictor variable. Among the predictive factors entered into the model, three factors with high regression coefficients were extracted to identify the determinants of happiness.

9. In conclusion, while the article contributes valuable insights into the relationship between leisure factors and adolescent happiness, addressing the highlighted weaknesses and implementing the recommended improvements would strengthen the overall quality and impact of the research.

Response: We did our best to address the highlighted weaknesses and implement the recommended improvements as stated above so that the overall quality and impact of the research can be improved.

Thank you so much for your kind and detailed comments.

All the best,

Eui Jae Kim, Hyun Wook Kang, Seong Man Park
