# Supplementary material for: Determinants of the happiness of adolescents: A leisure perspective
Source: PLoS One. 2024 Apr 9;19(4):e0301843. doi: 10.1371/journal.pone.0301843 (PMC11003699; doi:10.1371/journal.pone.0301843)
Supplement: Attachment — Submitted filename: Response to reviewers.docx [file pone.0301843.s004.docx]

Dear. Dr. Henri Tilga, Academic Editor

With regard to your revision request, we really appreciate your kind and strong support on the manuscript submission. As per your and reviewers’ revision request, we revised our manuscript so that the revised version of the manuscript fully addresses the points raised during the review process.

Here are the responses and revisions.

**Reviewer comments**

1. Include references in your R² and RMSE obtained values to validate with previous studies.
Response: We included the following references in our R² and RMSE obtained values to validate with previous studies.

Page 20: Hyper parameters were tuned to optimize the performance of the machine learning model. After hyper parameter tuning, the model's performance is shown in Table 6 and Figure 5. The highest-performing models were (R² = 0.121, RMSE = 1.315), Lasso (R² = 0.119, RMSE = 1.316), Linear Regression (R² = 0.115, RMSE = 1.319), Elastic Net model (R² = 0.108, RMSE = 1.325), XGBoost model (R² = 0.102, RMSE = 1.329), and LightGBM model (R² = 0.079, RMSE = 1.346). The performance of these models is somewhat low compared to previous studies that used machine learning to predict happiness [97-99].

Added references:

97. Airlangga G. Deciphering Urban Happiness: Analysis of Machine Learning Approaches for Comprehensive Urban Planning. Jurasik (Jurnal Riset Sistem Informasi dan Teknik Informatika). 2024; 9(1): 345-353.
98. Pan Z, Cutumisu M. Using machine learning to predict UK and Japanese secondary students’ life satisfaction in PISA 2018. Br J Educ Psychol. 2023;00: 1-25.
99. Prati, G. Correlates of quality of life, happiness and life satisfaction among European adults older than 50 years: A machine‐learning approach. ARCH GERONTOL GERIAT. 2022; 103: 104791.

2. After the previous arguments and after attending the recommendation, I recommend this paper to continue with the editorial process.

Response: We revised our reference list in order to ensure that it is complete and correct. We checked every single list one by one to check it is correct and it opens properly. We also checked the list to see we have cited papers that have been retracted, and we confirmed that there are no papers that have been retracted in the list. In the mean time, we found some typos and misinformation from the list, so we revised them one by one as follows:

13. Csikszentmihalyi M, Hunter J. Happiness in everyday life: The uses of experience sampling. J. Happiness Stud. 2003; 4: 185-199.

49. Chen Q, Chou CY, Chen CC, Lin JW, Hsu CH. The effect of leisure involvement and leisure satisfaction on the well-being of pickleball players. Sustainability. 2022; 14(1): 152.

70. Larson R, Csikszentmihalyi M. Experiential Correlates of Time Alone in Adolescence. J. Pers. 1978; 46(4): 677-693.

72. Toker B, Kalıpçı MB. Happiness among tourism students: a study on the effect of demographic variables on happiness. Anatolia. 2022; 33(3): 299-309.

83. Hakoköngäs E, Puhakka R. Happiness from nature? Adolescents’ conceptions of the relation between happiness and nature in Finland. Leis. Sci. 2023; 45(7): 665-683.

**Other changes**

1. We revised all the references in the list to conform to your reference formatting style.

2. In fig1, we deleted the red underlines (i.e., Multicollinearlity, XGBoost, Light GBM).

3. We found a typo (dada) in fig3 so we revised it correctly (data).

4. One author(Seong Man Park)’s affiliation has been changed from Department of General English Education, College of Libera Arts to Department of English Language, College of Foreign Languages), so we revised it accordingly.

Thank you so much for your kind and detailed comments.

All the best,

Eui Jae Kim, Hyun Wook Kang, Seong Man Park
